# Physical giftedness/talent: A systematic review of the literature on identification and development

**DOI:** 10.3389/fpsyg.2022.961624

**Published:** 2022-08-26

**Authors:** Jae Yup Jung

**Affiliations:** School of Education, University of New South Wales, Sydney, NSW, Australia

**Keywords:** physical, giftedness, talent, identification, development, systematic literature review

## Abstract

In this study, a systematic review was undertaken of the international scholarly literature on the identification and development of giftedness/talent in the physical domain, to establish the scope of current knowledge in the area. To identify relevant research, a search that involved the creation of a search string and the manual examination of the titles and abstracts of potentially relevant research, was conducted using two databases—*Web of Science Core Collection* and *SportDiscus*—and six inclusion/exclusion criteria (i.e., relevance to identification or development of physical giftedness/talent, an empirical study, publication in a reputable academic peer-reviewed journal, publication from 2000 to 2021, an English language publication, and authorship by scholars based in any part of the world). The 101 journal articles that met the inclusion/exclusion criteria were analyzed for key details, including the year of publication, methodological approaches, participants, and major findings. The five broad themes that emerged from the findings of these articles related to conceptions of physical giftedness/talent, identification characteristics/criteria, factors associated with identification, identification methods, and talent development interventions. An outline and discussion of the key issues and trends in the research, along with some recommendations for future research, conclude the systematic review.

## Giftedness and talent

For thousands of years, people have been fascinated by individuals who demonstrate exceptional abilities and accomplishments, commonly referred to as “gifted” and/or “talented” individuals (Subotnik et al., 2011). At the present time, no single definition or model of giftedness or talent has achieved wide acceptance among international scholars who study these individuals. Historically, the focus of the research on giftedness and/or talent has been on *intellectual* or *academic* giftedness/talent, and consequently, the definitions and models of giftedness/talent have been IQ-based, and consider the construct to be narrow, unitary, hereditary, and fixed (Terman, 1925; Terman and Oden, 1959). Nevertheless, more contemporary perspectives have adopted diversified definitions/models that better acknowledge the multiple possible domains (e.g., intellectual, creative, social, and physical) in which giftedness or talent may be seen. The focus of this systematic review of the literature is on giftedness or talent in the *physical* domain [i.e., any domain associated with, or related to, the human body, that may encompass strength, endurance, power, speed, flexibility, balance, agility, coordination, flexibility and fitness (Gagné, 2009; Farley et al., 2022)]. This is an area that appears to be a focus of investigation by sports scientists, but largely neglected by scholars in other fields with logical connections to physical giftedness/talent, including gifted education and psychology.

Within the field of sports science, a number of definitions of the construct have been proposed, invariably with respect to talent rather than giftedness. For example, Howe et al. (1998, p. 399–400) outlined a working definition of talent to be:

(1) It originates in genetically transmitted structures and hence is at least partly innate. (2) Its full effects may not be evident at an early stage, but there will be some advance indications, allowing trained people to identify the presence of talent before exceptional levels of mature performance have been demonstrated. (3) These early indications of talent provide a basis for predicting who is likely to excel. (4) Only a minority are talented, for if all children were, there would be no way to predict or explain differential success. Finally, (5) talents are relatively domain-specific.

In comparison, Brown (2002) proposed definitions for talent that refer to “a special, natural ability” or “a capacity for achievement or success”. Unfortunately, limitations exist with both definitions (Tranckle and Cushion, 2006). That is, the Howe et al. (1998) definition may be more accurately described as a *description* of the characteristics of talent in the physical domain, while the Brown (2002) definition may be overly generic and vague for useful practical application. As a result, some scholars have advocated for the adoption of models and definitions of giftedness/talent that originate from the field of gifted education, which give either implicit or explicit acknowledgment to giftedness/talent in the physical domain. Some of these models and definitions include:

(a) Renzulli's (1978, 1988) “three ring” model: Renzulli considers giftedness and talent to be synonymous terms that refer to the simultaneous possession or the capability to possess a cluster of three different traits—above average ability (top 15–20%) in any given area of human endeavor (i.e., including the physical domain), task commitment (i.e., motivation, perseverance, dedication, and self-confidence), and creativity (i.e., a focus on original thinking, curiosity, a willingness to take risks, or an openness to new ideas).
(b) Gagné's (2003, 2009) *Differentiated Model of Giftedness and Talent*: Gagné distinguishes between the phenomenon of giftedness (i.e., outstanding natural *abilities*) and talent (i.e., outstanding *achievements*), and proposes that giftedness may be translated into talent through a developmental process that may be influenced by intrapersonal factors, environmental factors, and chance.
(c) Subotnik et al.'s (2011, 2012) *Mega-Model of Talent Development*: Subotnik et al. (2011) propose that giftedness is a developmental construct that may be conceptualized differently according to the stage of engagement and development of an individual in a particular domain. In the initial stages, giftedness is primarily defined as ability, while in the later stages it is defined as achievement, and in the final stages of development, it is defined as eminence for exceptional individuals. The specific trajectory of the development of giftedness in this model (referred to as *talent development*) appears to be unique to each individual domain. Of relevance to physical giftedness, (Subotnik et al., 2011, p. 32) noted that:

“(w)hether a trajectory begins in early childhood or in adolescence, for example, depends on when the skills and abilities in the talent area emerge and coalesce …(which may be)… affected by physical maturation in fields such as music and sports … (and) … when talent can be recognized by systematic identification procedures”.

Of these models, Gagné's *Differentiated Model of Giftedness and Talent*, which formally defines giftedness as “the possession and use of outstanding natural abilities, called aptitudes, in at least one ability domain to a degree that places an individual at least among the top 10% of age peers” (Gagné, 2009, p. 63) and talent as “the outstanding mastery of systematically developed abilities, called competencies (knowledge and skills), in at least one field of human activity to a degree that places an individual at least among the top 10% of age peers who are or have been active in that field” (Gagné, 2009, p. 63), may have the greatest support to allow for the use of a common language in research and practice on giftedness/talent in the physical domain (Bailey and Morley, 2006; Tranckle and Cushion, 2006).

## Identification and development of giftedness/talent

Related to the issues around the conceptualization of giftedness/talent, there appear to be some lack of consensus on the identification and development of giftedness/talent in the physical domain. Indeed, scholars including Kozel (1996) and Tranckle and Cushion (2006), who are both in the field of sports science (and therefore refer to talent rather than giftedness), have noted the lack of agreement on theory and methodology relating to both talent identification and talent development. Nevertheless, talent identification appears to refer to the process of “recognition of individuals with potential to become elite” (Datson et al., 2020, p. 1313) or “potential to excel” (Norjali et al., 2018, p. 34). Some scholars have attempted to divide talent identification into a number of sub-categories that may be associated with its timing and the reference group. Specifically, Williams and Reilly (2000) have proposed the term talent detection to refer to the recognition of the strong potential of individuals who may not yet be involved in a particular sport, talent identification to refer to the recognition of the strong potential of current participants in the sport, and talent selection to refer to the ongoing process of recognition of strong potential at various stages of one's development in the sport.

Closely related to the identification of giftedness/talent in the physical domain is the development of such giftedness/talent, which is commonly understood to be the “provision of an optimal environment to realize this potential (to excel)” (Datson et al., 2020, p. 1313). Such environments may encompass various types of interventions, programs, or provisions, the composition of which may vary for each individual physical domain. Nevertheless, they appear likely to involve the development of the various combinations of physical, cognitive, social, and other characteristics and skills that may be necessary for success in each domain (Datson et al., 2020).

The purpose of identification and development of giftedness/talent in the physical domain has been variously described to include the development of an elite group of athletes (Datson et al., 2020), the promotion of national/team success (Toum et al., 2021), the efficient allocation of limited resources (Vaeyens et al., 2008), and financial rewards (Mann et al., 2017). To meet such objectives, the identification and development of physical giftedness/talent should ideally at least *commence* in school settings with generic identification and development tools that may be applicable across physical domains, as school settings may be optimal for maximum reach and inclusiveness of all children/ adolescents who have the necessary potential (Pion et al., 2015b). Nevertheless, much identification and development in the physical domain appears to take place within special programs organized by various bodies outside of schools, for reasons including the availability of expertise/resources, the lack of consensus on foundational constructs, and the lack of consensus on optimal approaches to identification and development (Prieto-Ayuso et al., 2022). As a result, there appears to be some lack of systematicity and comprehensiveness in the identification and development of giftedness/talent in the physical domain at the present time.

## Purpose of the study

The purpose of the study was to conduct a systematic review of the literature on physical giftedness/talent, with a particular focus on identification and development of physical giftedness/talent. More specifically, this study sought to outline the key details (i.e., study participants and methodological approaches) of the current research, summarize and synthesize the major findings of the current research, discuss the emerging issues and trends in the current research, and identify areas for possible attention in future research on the identification and development of physical giftedness/talent.

### Research question

The overall research question that guided the study was “*what is the state of empirical knowledge on the identification and development of giftedness/talent in the physical domain, according to the findings published in reputable English-language international peer reviewed journals from 2000 to 2021?*”

### Significance of the study

The study is significant, as it provides the first known systematic review of the literature on the identification and development of physical giftedness/talent. Related reviews of the literature have had a narrower focus, specifically on talent identification in sport or physical education (Johnston et al., 2018; Prieto-Ayuso et al., 2020). By providing an overview of the existing intellectual territory in the area (relating to a period of time that simultaneously captures the greatest portion of the research in the area, and the most recent period, and therefore the most relevant period, to inform future work), it is expected that this review will draw attention to the precise scope of existing knowledge and its strengths/ deficiencies, to set some clear directions for research and as a basis from which to move the area forward.

## Methods

### Search procedures

A search was conducted of the existing literature on the identification and development of giftedness/talent in the physical domain following key guidelines in the 2020 PRISMA (Preferred Reporting Items for Systematic Reviews and Meta-Analyses) statement (Page et al., 2021). The inclusion/exclusion criteria that directed the search were as follows:

(a) A study that is relevant to either the identification or development of giftedness/talent in the physical domain;
(b) An empirical study, defined as a study based on systematic observation and measurement of phenomena (Calfee and Chambliss, 2005), including studies that have focused on the secondary analysis of available data, but excluding reviews of the research, conceptual pieces, and opinion pieces on the identification/development of physical giftedness/talent;
(c) A study that is published in a reputable academic peer reviewed journal (i.e., an established journal that engages in a rigorous process of critical evaluation of the quality of studies, involving experts in the field, prior to publication), and therefore excludes research that is published in publication outlets other than peer reviewed journals such as conference proceedings, books, book chapters, reports, government documents, and dissertations, which may undergo less rigorous review procedures (Borrego et al., 2014);
(d) A study that is published in the period from January 2000 to August 2021 (including advance online publications that were available during this period);
(e) A study that is published in the English language; and
(f) A study that is published by researchers based in any part of the world.

As the first step, these inclusion/exclusion criteria were applied to two databases that together give strong coverage to physical giftedness/talent—*Web of Science Core Collection* and *SportDiscus*. The Web of Science Core Collection is a set of multidisciplinary databases that includes the Science Citation Index Expanded, the Social Sciences Citation Index, the Arts and Humanities Citation Index, and the Emerging Sources Citation Index, while SportDiscus is recognized as the leading bibliographic database for sports and sports medicine research (EBSCO, n.d.).

### Web of Science Core Collection/SportDiscus

In conducting the search in the *Web of Science Core Collection* and *SportDiscus* databases, a number of search terms were combined into a search string:

[gift^*^ OR talent^*^ *OR high potential OR high ability] AND physical AND [identification OR selection OR assessment OR program OR intervention OR development]*

**Figure 1:**
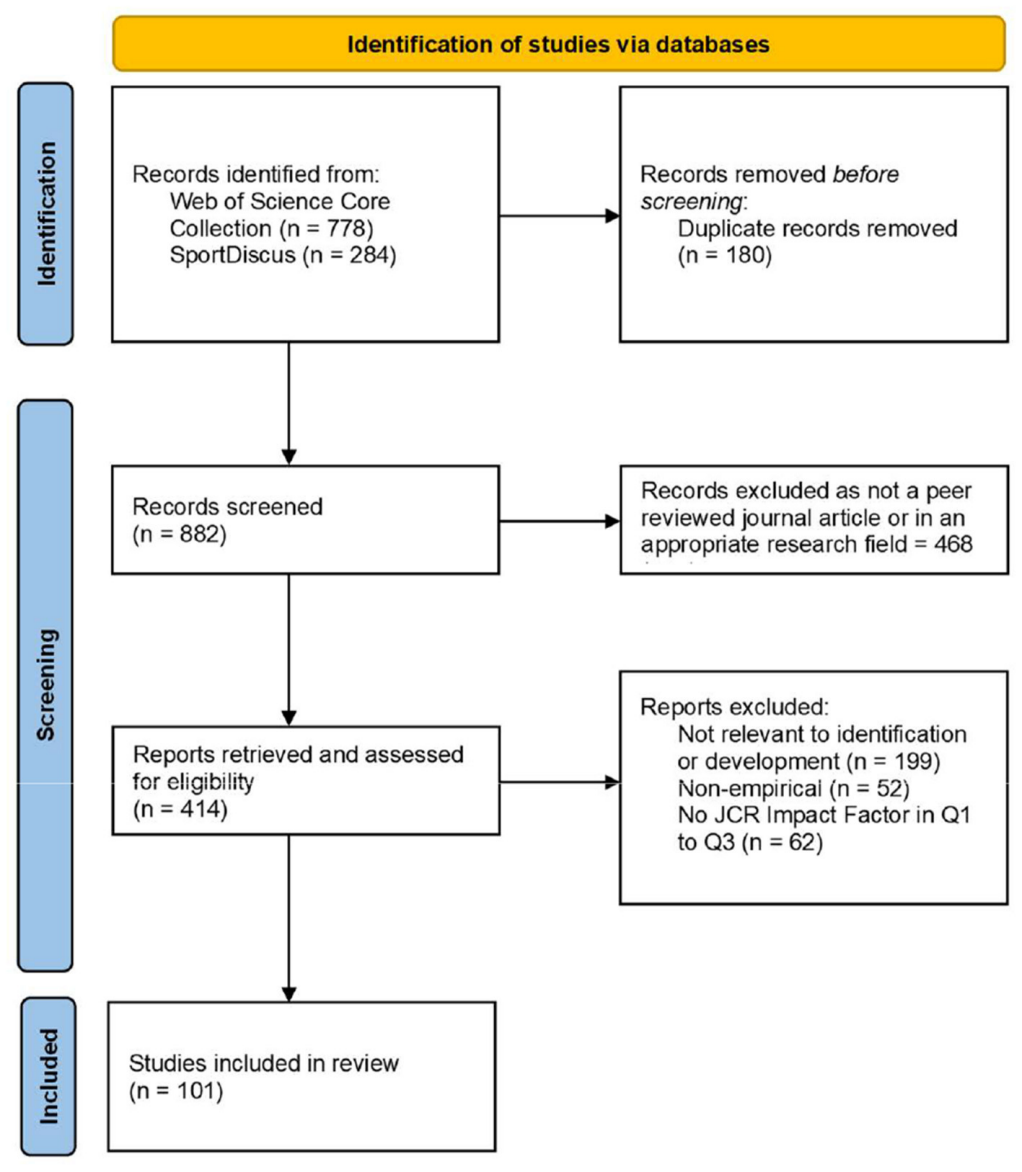
PRISMA flow diagram of the search process.

First of all, this search string was used to investigate the titles and abstracts of all English language publications listed in the two databases from January 2000 to August 2021. This procedure resulted in the identification of 778 items in the *Web of Science Core Collection* and 284 items in *SportDiscus*. After the removal of 180 duplicate items, a total of 882 items remained in the two databases. Thereafter, any items that did not qualify as a peer reviewed journal article (i.e., proceedings papers, book chapters, data papers, and retracted publications) or were classified as being in an irrelevant research area (e.g., Engineering, Environmental Sciences, Food Sciences Technology, and Chemistry) were removed, to leave 414 items for further consideration. The titles, abstracts, and if necessary, other relevant sections, of the 414 items were manually examined to confirm their relevance to the identification or development of physical giftedness/talent, and the empirical nature of the study. During this process, “identification,” “development,” and “physical giftedness/talent” were broadly defined, and therefore allowed for the inclusion of articles that, for example, gave coverage to the non-physical development of physically gifted individuals (e.g., development of mental health), and giftedness outside of sports (e.g., dance). It is noted that multiple articles needed to be removed at this stage, as they represented proposals or reports of talent development programs that did not qualify as empirical studies. The procedure resulted in the removal of a further 251 items, leaving 163 items for further consideration. Finally, to ensure that only articles of a sound quality and rigor were included in the review, only those articles that were published in journals that had a Journal Citation Report Impact Factor in the first to third quartiles in any relevant field (e.g., Sport Sciences, Education and Educational Research, Psychology Multidisciplinary, and Applied Psychology) in the year of publication were retained. This left a total of 101 English language peer reviewed journal articles that met all of the inclusion criteria to form a part of the systematic literature review (details of these journal articles appear in the Supplementary material). Figure 1 outlines a PRISMA flow diagram that provides greater details of the search process.

### Analysis

The 101 articles that were determined to be part of the systematic literature review were analyzed by coding with respect to the year of publication, the methodological approaches that were adopted, and the nature of the participants. Furthermore, to inform and organize the summary/synthesis of the key findings of the selected articles, thematic analysis was undertaken of those parts of the abstracts relating to the findings of these 101 articles, following the guidelines of Braun and Clarke (2006). The specific analytic procedure that was adopted was a recursive and iterative process of: (a) familiarization with the data through multiple readings of the abstracts, (b) the generation of codes, which are the most basic segments of data relating to the key findings of each study, by asking questions including “What is being described in the data?”, (c) the identification of potential themes through the sorting and grouping of codes, (d) the review of each theme for internal coherence and distinctness from other themes, and (e) the identification of broad themes that involve the sorting and grouping of each of the identified themes (Attride-Stirling, 2001; King, 2004). An inductive/semantic, rather than an interpretative, approach to thematic analysis was adopted, to ensure that the final thematic structure closely reflected the data on the key findings of the selected studies. Moreover, in arriving at the names for each code/theme/broad theme, efforts were made, as much as possible, to reflect the raw data (Boyatzis, 1998). Finally, at the conclusion of the thematic analysis, the final thematic structure was reviewed to confirm that it did indeed reflect the key findings of the 101 selected studies, through a final review of the abstracts of all selected studies. Table 1 outlines the final thematic structure comprising 154 codes, 61 themes, and 5 broad themes (i.e., conceptions/domains of physical giftedness/talent, identification characteristics/criteria, factors associated with identification, identification methods, and talent development interventions).

**Table 1:** Major topics of identified articles.

**Broad theme**	**Theme**	**Code**
Conceptions/domains of physical giftedness/talent	Conceptions	Conceptions of ability in PE, definitions, talent in PE, talent in sport
	Dance	Dance
	Disability	Para
	Sports	Alpine skiing, archery, athletics, Australian football, badminton, baseball, basketball, cricket, fencing, figure skating, futsal, golf, gymnastics, handball, hockey, ice hockey, judo, netball, rugby league, rugby union, soccer, swimming, table tennis, taekwondo, tennis, triathlon, volleyball, water polo
	Teacher perceptions	Teacher perceptions, coach perceptions
Identification characteristics/criteria	Age	Age
	Anthropometrics	Anthropometrics, body size
	Changes in performance characteristics	Changes in performance characteristics, changes to physical fitness characteristics, reliability, stability
	Cognitive-motor skills relationship	Cognitive-motor skills relationship
	Developmental level	Developmental level, junior/senior level
	Female	Female
	Game-based performance indicators	Game-based performance indicators
	Identification criteria	Identification factors, talent selection criteria
	Motor coordination characteristics	Motor characteristics, motor coordination
	Performance characteristics	Performance characteristics
	Performance level	Competition level, selection level, performance level, team success
	Physical performance characteristics	Physical characteristics, physical performance characteristics, endurance capacity, physical fitness, fitness variables, physiological characteristics, locomotor characteristics, athletic movement, physical ability, physical profile
	Playing position	Playing position
	Predictors of success in sport-specific skills	predictors of freethrow effectiveness, predictors of repeated sprint ability, predictors of vertical jump performance
	Profiles	Profiles
	Psychological characteristics	Psychological characteristics
	Sport-specific skills	Ball handling, fast bowling, estimation of attacking range, reachability, vertical jump, skills
	Tactical skills	Decision-making, game intelligence
Factors associated with identification	Biological maturation	Biological maturation
	Coach efficacy expectations	Coach efficacy expectations
	Draft selection	Draft selection, draft selection order
	Genetics	Genetics, genotype
	Predictors of career success	Prediction of professional contract, prediction of future international squad selection, prediction of medal success, predictors of career success, predictors of national team selection, predictors of representative selection, predictors of squad selection, prediction of future draft order
	Predictors of future performance	Predictors of future performance, predictors of march performance, predictors of performance, predictors of playing time, predictors of playing potential
	Previous match physical performance	Previous match physical performance
	Professional success	Progression to professional, professional career attainment
	Progression in training	Continuation of training, progression in youth sport, player retention
	Relative age effect	Relative age effect, reverse relative age effect
Identification methods	Agreement between identification instruments	Agreement between identification instruments
	Bio-banding	Bio-banding
	Identification batteries	Non-sports specific generic testing battery, non-sport-specific motor test battery, talent identification battery, testing battery
	Identification instruments	Judo-specific test, objective assessments, subjective assessments, endurance field tests, motor tests, psychological characteristics instrument, small sided games kicking proficiency assessment, physical draft camp tests
	Identification ratings	Advanced players, coaches, novice players
	Multidimensional assessment	Multidimensional assessment
	Performance appraisal interview	Performance appraisal interview
	Sports classification	Sports classification
Talent development interventions	Accountability	Accountability
	Age-related performance trajectories	Age-related performance trajectories
	Early diversified sports participation	Early diversified sports participation
	e-mentoring	E-mentoring
	Expertise acquisition	Expertise acquisition
	Feedback	Feedback
	Mental health	Mental health
	Motivation	Motivation, will to compete, will to excel
	Non-development of talent	Non-development of talent
	Peer assessment	Peer assessment
	Physical education	Physical education
	Planned disruptions	Planned disruptions
	Play	Play
	Practice	Practice
	Program efficacy	Program efficacy, training response
	Reinforcement	Reinforcement
	Skill-based training program	Skill-based training program, skills-based coaching intervention
	Sport-specific training	Small-sided games, soccer-specific training
	Talent development processes	Talent development processes
	Talent development program	Talent development program

A quality assessment of the systematic review of the literature, undertaken with the *CASP–Systematic Review* tool (Critical Appraisal Skills Program, 2022), demonstrated that the review was undertaken in a rigorous manner.

## Results

### Study participants

The participants of the 101 studies included in the review mostly comprised young gifted/talented athletes in various sports (e.g., soccer, Australian football, handball, volleyball, gymnastics, rugby league, and ice hockey), who qualified as being gifted/talented due to criteria such as selection into talent development programs, invitation to selection trials for representative squads, regional/national/international representation, high regional/national ranking, selection for training camps, strong competition results, and membership of an academy or professional club. Often, the studies made comparisons between gifted/talented athletes of different competitive/performance levels (e.g., national representatives vs. club membership), or between gifted/talented athletes of varying developmental levels (e.g., under 13 vs. 15 years or adolescent vs. adult athletes). Female gifted/talented cohorts were a focus of 10 studies. Eleven studies had a focus on elite senior athletes, rather than young developing athletes (usually described as “adolescents,” “youth,” or “junior” athletes), which tended to concentrate on the anthropometric and physical performance characteristic profiles of these athletes that may be used as benchmarks for talent identification and development. Among those studies that had non-athletes as participants were studies that had coaches (nine studies), physical education teachers (three studies), clinicians (one study), undergraduate students studying physical education (one study), and a panel of sports experts (one study), who may all be considered stakeholders in the identification and/or development of those who are physically gifted or talented. Finally, three studies focused exclusively on the secondary analysis of pre-existing data (i.e., birth dates, race times, and physical performance data).

### Method/methodology

Both quantitative (89 studies) and qualitative (9 studies) research methodologies were employed in the studies that formed a part of the systematic literature review, along with a few studies (3 studies) that combined these two approaches. The key methods used to collect data for these studies included physical performance assessments (70 studies), anthropometric assessments (55 studies), sports-specific technical assessments (19 studies), motor co-ordination assessments (15 studies), biological maturity assessments (15 studies), interviews (11 studies), and surveys/questionnaires/scales (11 studies).

### Key findings

The findings of the 101 selected studies related to five areas that reflected each of the five broad themes that emerged from the thematic analysis—conceptions and domains of physical giftedness/talent, the characteristics/criteria used to identify giftedness/talent in the physical domain, other factors associated with the identification of physical giftedness/talent, the methods that may be used to identify physical giftedness/talent, and interventions that may be used to develop talent in the physical domain. The following provides a general summary and synthesis of each of these areas, after a thorough review of the relevant articles associated with each area. It is noted that there was some inevitable overlap between some of these broad areas (e.g., some performance characteristics may not only be considered identification characteristics/criteria but also benchmark indicators associated with, or which may be used to inform, talent development interventions), and that it was not possible or appropriate for the key findings of all 101 articles to be outlined in the summary/synthesis.

#### Conceptions/domains of physical giftedness/talent

Two studies outlined empirical findings relating to conceptions of giftedness/talent in the physical domain. Of note, both studies use the term “talent” rather than “giftedness” to refer to excellence in the physical domain, which was also the case in most of the other selected 101 studies. Interestingly, both of these studies are set in physical education contexts. The focus of Croston (2013) was on investigating the perceptions of physical education teachers on talent in physical education and sport, within the context of directives in English policy that appear to have merged the goals of physical education and sport. She found that the participating physical education teachers distinguished, to various degrees, between talent in physical education (associated not only with physical ability, but also with personal health, social, creative, and cognitive abilities) and talent in sport (associated predominantly with physical excellence that may be directly observed or measured). The distinction between the constructs appears to reflect the view that physical education should be guided by educational objectives to support the talent development of all students, while sport has a greater focus on elite performance and competition.

In comparison, Hay and Macdonald (2010) investigated the existence or otherwise of empirical support for the social construction of physical ability (Evans, 2004), which represents a departure from traditional perspectives that view ability from a scientific/biological perspective, acknowledge its genetic components, and consider it to be something that may be identified through a battery of measurements focusing on the anthropometric, physiological, and psychological characteristics. Through analysis of physical education curricula, interviews, and observations of teachers and students, the authors demonstrated support for the idea that physical ability may also be influenced by an individual's cultural and social capital, available resources and opportunities, and the mechanisms by which the value of, and recognition for, ability are established.

Among other studies, Hogarth et al. (2021) does not have a focus on conceptualizations of physical giftedness/talent, but is one of two studies with a focus on Paralympic athletes (i.e., the age-related performance trajectories of Paralympic swimmers), and therefore acknowledges an expanded conceptualization of physical giftedness/talent to include those who simultaneously have a physical, visual, or intellectual impairment. The other study to acknowledge twice exceptionality (i.e., the simultaneous possession of giftedness/talent and a disabling condition) was Spathis et al. (2015), which tested the psychometric properties of a talent identification instrument for five different categories of Paralympic throws (i.e., seated and standing javelin, shot put, discus, and seated club throws).

Irrespective of the specific conceptions of physical giftedness that formed the basis of the 101 selected studies, the different *domains* of physical giftedness/talent covered in these studies were, in order of frequency, soccer, Australian football, handball, volleyball, gymnastics, rugby league, ice hockey, tennis, basketball, fencing, rugby union, water polo, field hockey, alpine skiing, athletics, cricket, dance, figure skating, futsal, golf, judo, netball, swimming, taekwondo, baseball, badminton, table tennis, triathlon, and archery.

#### Identification characteristics/criteria

The vast majority of the studies related to the characteristics and/or criteria for the identification of physical giftedness/talent provided anthropometric and physical performance (and to a lesser extent, motor co-ordination) profiles of young gifted/talented adolescents or children in a wide range of sports. The anthropometric profiles were usually arrived at using measurements of standing height and body mass, while the physical performance profiles (encompassing physical fitness profiles, and physiological profiles) typically incorporated assessments of speed, flexibility, muscle strength, endurance, and/or muscle power. The motor co-ordination characteristic profiles, when investigated, were usually assessed using the Körperkoordinations Test für Kinder (KTK; Kiphard and Schilling, 2007), which is an instrument that comprises subtests in walking backwards, moving sideways, jumping sideways, and hopping for height.

These studies commonly provided the anthropometric/ physical/motor co-ordination profiles of a target group of gifted/talented young adolescents or children in comparison to equivalent profiles for one or more comparison or reference groups (Ransdell and Murray (2011), Mkaouer et al. (2018), and Nassib et al. (2020) were exceptions that focused on a single cohort). Such comparison groups usually comprised those of a different performance/competitive level (e.g., non-elite, league players, and international representatives in handball in Moss et al. (2015); medalists and non-medalists at national youth fencing championships in Norjali et al. (2018)), or different developmental levels (e.g., youth, academy, and senior rugby league players in Dobbin et al. (2019); U16 and U18 Australian football players from a state academy in Gaudion et al. (2017)). Some studies included both types of comparison groups (e.g., Vaeyens et al., 2006; Farley et al., 2022). Typically, these studies found that the children and adolescents who performed at higher performance/selection levels or were at higher developmental levels tended to have superior characteristics on *some* anthropometric, physical performance, or motor co-ordination assessments, depending on the particular sport and performance/developmental level. For example, Matthys et al. (2011) found that in comparison to their peers at lower performance levels, junior handball players who performed at the highest levels demonstrated significantly greater aerobic capacity, strength, power, speed, and agility, after controlling for maturation. Among those studies that investigated the anthropometric/physical/motor co-ordination profiles of cohorts at different developmental levels, the differences in the profile characteristics lead to a common conclusion about the age-dependent nature, and the instability over time, of the factors associated with talent identification (Vaeyens et al., 2006; Buchheit and Mendez-Villanueva, 2013; Nikolaidis et al., 2015; Woods et al., 2016a, 2017; Bidaurrazaga-Letona et al., 2019).

Rather than making comparisons between gifted/talented youth and those of different performance or developmental levels, a number of studies focused on making comparisons of the anthropometric and physical performance profiles of gifted youth for different *playing positions* in team sports. These studies included findings that in rugby union, U18/U19 forwards differ from backs in their lower countermovement jump heights, lower triple hops, and 10 m higher sprint times (Wood et al., 2018), while at the adult professional level, forwards were heavier and taller and have a larger percentage body fat and fat-free mass than backs (Fontana et al., 2015). In comparison, Nikolaidis et al. (2015) found that adult wings in handball differed considerably from the other positions in having a smaller body mass, height, fat-free mass, and anaerobic power, although such characteristics did not differ significantly among adolescent wings and other positions.

Considerably less attention appears to have been devoted to the investigation of the *other* characteristics of gifted/talented individuals in the physical domain, including sports-specific skills, perceptual-cognitive/tactical skills, and psychological skills. Nevertheless, a few studies investigated a mix of these characteristics. For example, Woods et al. (2016b) found that in Australian football, U18 state representatives outperformed non-representatives in a multi-dimensional assessment that incorporated anthropometric and physical performance assessments (i.e., standing height, dynamic vertical jump height and 20 m multistage fitness test), along with sport-specific (i.e., kicking and handballing) tests and perceptual-cognitive tests relating to decision-making.

Some sports-specific skills that have been separately investigated in the literature include skills associated with fencing, cricket, and soccer. Specifically, Turner et al. (2017) suggested that the most talented fencers may be those who have the greatest accuracy in predicting their attacking range (e.g., lunging and step lunge distances), which may in turn be influenced by anthropometric characteristics. In comparison, Phillips et al. (2014) found that among the factors that may be early markers of potential in cricket fast bowling may be a high level of intrinsic motivation (i.e., “fun” and “enjoyment”) and general skills associated with fast bowling in cricket. In youth soccer, Scharfen and Memmert (2019) identified relationships between superior cognitive characteristics in attention/working memory and dribbling, ball control and ball juggling, which may all be considered to be essential skills for success in soccer.

The literature provides somewhat mixed findings on the relevance of psychological characteristics, such as those relating to motivation, in the identification of physical giftedness/talent. For example, in dance, Aujla et al. (2015) found that lower levels of ego-involving motivational climate perceptions (i.e., a perception that instructors provide selective praise and punishment, and a focus on superior performance and objective talent) and greater levels of harmonious passion (i.e., flexible activity involvement where the individual participates in the activity on his or her own volition) may be conducive to a greater likelihood of continuation in training and development. In contrast, Matthys et al. (2011) was not able to identify a difference between club players and those who had higher representation in youth handball, in terms of task or ego motivational orientation. With respect to psychological constructs that are unrelated to motivation, Towlson et al. (2022) suggested that characteristics such as confidence, competitiveness, and a good attitude may be key in the identification process.

#### Factors associated with identification

A group of studies that utilized retrospective or longitudinal research designs investigated those characteristics of gifted/talented individuals in the physical domain that may be predictive either of future success at the senior level (e.g., the award of professional contracts) or retention/continuation in the talent development pathway. Effectively, these studies allowed for an understanding of those characteristics that may be conducive to the transformation of high potential in the physical domain into corresponding future achievements. Collectively, these studies produced mixed findings.

A small number of studies, including Craig and Swinton (2020) and Cripps et al. (2020), relating to soccer and Australian football, respectively, indicated that while those young adolescents who do eventually achieve future success have (on average) slightly superior anthropometric and physical profiles than those who are less successful, these differences may not be substantial enough to reliably predict future career attainment within an already talented pool of gifted/talented athletes. In contrast, a number of other studies have suggested that possible predictors of future success may indeed exist, with speed, aerobic capacity, and motor coordination commonly noted across sports. For example, speed, aerobic endurance, motor co-ordination, and agility have been suggested to be among the optimal predictors of professional career attainment in male soccer (Deprez et al., 2015; Dugdale et al., 2021; Patel et al., 2021), while speed and the percentage of time spent sprinting and the number of sprints per minute during match play may be predictive of career success in Australian football (Burgess et al., 2012). In gymnastics, strong basic motor skills, shoulder strength, leg strength, and gross motor coordination appear to be critical (Pion et al., 2015c). The age dependency and the non-linear nature of the development of some of these predictors has been highlighted by scholars in the area—for example, Dugdale et al. (2021) noted that those who enter the talent development pathway before 13 or 14 may be substantially less likely (than those who enter from age 13 or 14) to achieve professional career success, while Deprez et al. (2015) proposed that motor co-ordination, speed, and aerobic endurance may be important in soccer prior to and during one's age at peak height velocity, and explosive power may be significant after the age at peak height velocity. The variation in the findings of these studies may reflect the sports-specific nature of the predictors of future success.

A separate group of studies investigated the phenomenon of biological maturation. Biological maturation refers to changes in body dimensions and hormonal profiles during adolescence that coincide with significant improvements in strength, speed, and power (Malina et al., 2015). Multiple studies have identified the transient advantages that biological maturation (alternatively referred to as skeletal and somatic maturation) have on the anthropometric and physical performance characteristics of the affected individuals, which may serve to inform stakeholder perceptions of the potential of these individuals, and confound the identification process (Furley and Memmert, 2016; Till et al., 2017; Towlson et al., 2017; Peña-González et al., 2021; Toum et al., 2021). That is, early and advanced maturers may be preferentially identified as gifted or talented in the physical domain, and consequently gain preferential access to high level training opportunities. To address such effects, scholars have suggested the potential benefits of making comparisons of performance on the basis of maturity rather than chronological age (Peña-González et al., 2018; Lovell et al., 2019), the introduction of maturity-based quotas in identification (Lovell et al., 2019), the incorporation of assessments which may not be strongly influenced by biological maturation (e.g., motor competence and sports-specific skills) into identification processes (Toum et al., 2021), and the establishment of talent development pathways that are targeted specifically at late maturers (Myburgh et al., 2016).

Closely related to, but not synonymous with, biological maturation is the relative age effect, which refers to the impact of the timing of one's birth within a chronological age category on the identification of giftedness or talent (Toum et al., 2021). As for biological maturation, the relative age effect appears to confound the identification process, as those who are born earlier tend to be substantially advantaged. Evidence of the relative age effect has been seen in multiple sports that have a disproportionately large number of athletes born in the first quartile of each age category identified for representative teams/talent development programs (Helsen et al., 2005; Votteler and Höner, 2014; Gorski et al., 2016). Scholars have suggested that the phenomenon may arise due to stakeholder expectations about those who are relatively older, along with the traditional reliance on anthropometric and physical performance characteristics in the identification process (Helsen et al., 2005; Peña-González et al., 2018). Many of the recommendations that have been proposed to address the relative age effect are similar to the recommendations to address the effects of biological maturation—a greater focus on longer term performance in the identification process (Helsen et al., 2005; Coutts et al., 2014; Andronikos et al., 2016), the education of relevant stakeholders about the relative age effect (Coutts et al., 2014; Andronikos et al., 2016), the avoidance of early identification (Andronikos et al., 2016), separate talent development provisions for those who are relatively younger (Coutts et al., 2014; Andronikos et al., 2016), the normalization of performance indicators with respect to age, body mass, or height (Gorski et al., 2016; Peña-González et al., 2018), greater emphasis on technical skill assessments which may be less influenced by relative age (Votteler and Höner, 2014; Andronikos et al., 2016), the creation of smaller chronological age categories (Helsen et al., 2005), and the rotation of the cut-off dates that define each chronological age category (Helsen et al., 2005).

Finally, the findings appear mixed with respect to the usefulness of genetic testing for talent identification. On the one hand, Znazen et al. (2016) proposed that one's genetic background may play an important role in sporting potential and may cause some individuals to be better adapted to specific physical training. On the other, Suraci et al. (2021) refuted the possible usefulness of genetic testing for the assessment of athletes or the identification of talent.

#### Identification methods

The studies associated with specific *methods* of identification each had a slightly different focus. Nevertheless, many of these studies indicated the importance of a *multiple criteria* or a *multidimensional* approach to identification of giftedness/talent in the physical domain. For example, Dugdale et al. (2020), noted that while there may be some alignment between the outcomes of the objective and subjective approaches to the identification of physical fitness for the highest and lowest performers in youth soccer, such alignment is less likely among those whose performances are not so distinct, and therefore advocated for identification processes that utilize both objective and subjective assessments. In comparison, Dobbin et al. (2019) while reporting on a study that demonstrated the usefulness of a standardized testing battery that differentiates between the anthropometric/physical performance characteristics of rugby league players of different developmental levels, noted that a limitation of the battery was its lack of assessment of other variables (i.e., technical, tactical, social, and psychological variables) that may be necessary for successful match performance. These studies highlight the importance of collecting data from multiple sources and instruments to maximize the effectiveness of identification.

The other studies relating to identification methods had a narrower focus on the individual types of identification instruments, individual identification instruments, and individual aspects of the identification process. For example, both Vandorpe et al. (2012) and Pion et al. (2015b) investigated the usefulness of non-sports specific assessment batteries that may form a part of a larger identification process. Specifically, Pion et al. (2015b) noted that the *Flemish Sports Compass* (comprising 22 tests of anthropometrics, physical performance, and motor co-ordination), which classifies young athletes across nine sports, may be useful in directing young children toward sports that suit their individual characteristics. In comparison, Vandorpe et al. (2012) noted the superiority of a generic non-sports specific motor test battery (i.e., the KTK) to coach judgments, anthropometric tests, and physical performance tests, in the prediction of the future success of female gymnasts. The authors attributed the lack of reliability and stability of anthropometric and physical performance assessments to the fact that they are likely to be affected by factors including growth, maturation, and training.

Sports-specific identification instruments were the focus of a number of other studies. For example, Lidor et al. (2005) assessed the usefulness of a judo-specific ability test (comprising ten stations that each assessed a physical ability or skill) for its prediction of future performance, and concluded that its limited usefulness may be associated with its non-assessment of skills that are necessary in authentic match environments. In comparison, Bonney et al. (2020) outlined the development and psychometric testing of an Australian football kicking assessment, which was demonstrated to be valid and reliable in the discrimination of skill levels in kicking performance. The general validity and reliability of a sports-specific identification instrument was also confirmed by Spathis et al. (2015), who assessed a talent identification battery for five Paralympic throws.

The studies that focused on the *non-physical* identification methods included MacNamara and Collins (2011) and Kilger and Jonsson (2017). MacNamara and Collins (2011) outlined the development and validation of a psychological characteristics questionnaire (i.e., *Psychological Characteristics of Developing Excellence*), which not only assessed the *possession* of psychological characteristics that may be associated with talent development, but also the *use* of such characteristics for talent development. In comparison, Kilger and Jonsson (2017) conducted a study on performance appraisal interviews during progression in a talent pathway. They proposed that for continued selection as an elite athlete, gifted/talented athletes require not only strong levels of physical performance, but also strong social interaction skills (e.g., the demonstration of gratitude for training opportunities, high self-esteem and confidence about one's current development status, the setting of high goals for one's future development, and humility) that may be assessed during performance appraisal interviews.

Finally, Towlson et al. (2022) conducted a study with a focus on bio-banding, which is an alternative to chronological age grouping, and involves the grouping of players on the basis of maturity status, for the purposes of identification. Bio-banding is a strategy that is designed to address the confounding effects of biological maturation and the relative age effect on the identification process. Of the various approaches to bio-banding, two of the most common appear to be the “percentage of estimated adult stature attainment” method and the maturity offset method (which models the normal growth curves of adolescents with an individual's anthropometric characteristics), which each have strengths and weaknesses. A key finding in Towlson et al. (2022) was the usefulness of maturity mismatched bio-banding small-sided games in allowing the less physically mature soccer players to display important psychological characteristics.

#### Talent development interventions

Many of the abovementioned studies that outlined the anthropometric, physical performance, motor co-ordination, sport-specific skill, and psychological characteristics of gifted/talented youth that are relevant to identification, may also be considered relevant to the development of talent development interventions, as they may inform the specific foci of such interventions, and/or as benchmarks to monitor developmental progress (Farley et al., 2022). For example, from a talent development perspective, the anthropometric and physical performance characteristics that have been recognized to be correlated with golf performance in Wells et al. (2009) provided a rationale for golf training programs that promote flexibility, balance, core strength, upper/lower body strength and power, and cardiovascular conditioning. Similarly, the age-related performance characteristics and trajectories of Paralympic swimmers in Hogarth et al. (2021) provided useful benchmark performances linked to age, sex, and disability classification that may allow for the setting of meaningful training goals.

A group of studies that did *not* profile the performance characteristics of gifted/talented youth, had a focus on talent development interventions that may apply to gifted/talented youth across multiple physical domains *within school settings*. Specifically, in Collins et al. (2010), the efficacy of an educational program designed to promote lifelong physical activity and talent development in children (i.e., *Developing the Potential of Young People in Sport*), involving formal lessons and participation in extracurricular activity clubs, was demonstrated through post-program improvements in activity levels and enhancements to perceived competence and self-determination. In comparison, Prieto-Ayuso et al. (2022) outlined the possible barriers for teachers in fully supporting talent development of gifted physical education students (i.e., a lack of curricular guidelines, a lack of knowledge about useful interventions, a lack of time, and a mentality that appropriate interventions are provided outside of school), which may inform measures to support such students in the future. One recommendation to overcome the identified barriers was the provision of more effective teacher professional development.

A number of other studies provided findings relating to talent development interventions *outside* of school settings that may arguably have application across multiple sports. For example, Kegelaers et al. (2020) interviewed expert coaches in high performance sports on the use of planned disruptions (i.e., structured and deliberate training activities where athletes are exposed to increased and changing demands), to find that they exist in nine broad forms (i.e., location, competition simulation, punishments/rewards, physical strain, stronger competition, distractions, unfairness, restrictions, and “outside the box”). The participating coaches believed that the benefits of planned disruptions largely lie in the increased familiarization with pressure, the greater awareness of one's thoughts/behaviors in such situations, the development of personal resources, and the promotion of team processes such as connectivity and leadership. In comparison, Holt et al. (2012) noted the possible benefits of talent development interventions that incorporate self-set goals, peer assessments of performance, and group rewards for reaching personal goals, due to the improvements that were seen in the level and consistency of performance on targeted skills when the interventions were implemented. In contrast, Hendry et al. (2018) identified the greater benefit of high quality, structured “practice” activities to unstructured “play” activities for skill development.

Complementing such studies are multiple studies related to talent development interventions that may only apply to individual sports. Among these are studies that demonstrated: (a) the efficacy of small-sided games in the development of biomotor abilities (i.e., speed, agility, power, and aerobic capacity) in soccer (Suraci et al., 2021), (b) the usefulness of participation in diverse sports during one's childhood in the development of movement skills for basketball (Arede et al., 2019), (c) the factors that may be predictive of the acquisition of expertise in fast bowling in cricket (i.e., intrinsic motivation in early development, and a good attitude, pace of bowling, bowling technique, co-ordination, and training ethic in later development (Phillips et al., 2014)), and (d) the effectiveness of an 8 week skill-based volleyball training program in the enhancement of skills in passing, setting, serving, spiking, and blocking (Gabbett et al., 2006).

A final group of studies investigated talent development interventions that are not “physical” in nature, but nevertheless have significant consequences on physical performance. One of these related to the mental health of young athletes, which may be evidenced by sudden behavioral changes, along with behaviors associated with anxiety, depression and perfectionism, and may be related to risk factors such as an unstable family or “pushy” parents, or a non-supportive performance environment (Hill et al., 2016). Among the recommendations of Hill et al. (2016) were that coaches and other relevant support staff need to be provided with training on how to deal with mental health issues, the promotion of mental health awareness among families, and the development of assessment tools to flag potential mental health issues. In comparison, Gonçalves et al. (2014) highlighted the need to pay attention to motivational factors (i.e., the will to excel and the will to compete) during talent development programs, while Lamb and Aldous (2014) noted the benefits of e-mentoring for gifted physical education students as a means to discuss possible problems, to gain information and advice on relevant opportunities, and to manage injuries.

## Discussion

### Overview of key findings

Table 2 provides an overview of the 10 key findings published in reputable English language, international peer reviewed journal articles on the identification and development of giftedness/talent in the physical domain, from 2000 to 2021.

**Table 2:** Key findings on the identification and development of giftedness/talent in the physical domain.

1. The term “talent” is substantially more commonly used than “giftedness” to refer to exceptional ability and achievement in the physical domain
2. Conceptions of talent in the physical domain appear to rely mostly on scientific/biological perspectives
3. A distinction between talent in physical education and talent in sport have not always been reflected in policy developments with respect to talent development in individual countries
4. Multiple anthropometric, physical performance, and (to a lesser extent) motor co-ordination profiles have been developed of young gifted/talented athletes across a range of sports, often in comparison to others of different developmental levels and/or performance/competitive levels, or with respect to individual playing positions in team sports
5. Although dependent on age and the particular sport, characteristics such as speed, aerobic capacity, and motor coordination are often recognized as being predictive of future success at the senior level.
6. Two related phenomena—biological maturation and the relative age effect—may confound the talent identification process
7. A comprehensive multidimensional approach to talent identification, that incorporates objective and subjective assessments, along with assessments of the full range of performance characteristics (e.g., anthropometric, physical performance, motor coordination, sports-specific technical skills, cognitive, psychological, tactical, and social) is ideal
8. A number of non-sport specific (e.g., *KTK*) and sport specific (e.g., *Australian football kicking assessment*) identification instruments, and approaches to identification (e.g., bio-banding), have been demonstrated to be useful in the identification of giftedness/talent in the physical domain.
9. A range of non-sport specific (e.g., *Developing the Potential of Young People in Sport*) and sport specific (e.g., small-sided games in soccer) talent development interventions have been demonstrated to be effective for gifted/talented athletes in the physical domain
10. Important talent development interventions outside of the sports field may include programs to support the mental health and motivation of athletes, possibly with the involvement of mentors

### Key issues and trends in the research

A number of key accomplishments have been seen in the research on the identification and development of physical giftedness/talent in the investigated period. These include: (a) the increasing acknowledgment of diverse types of performance characteristics in the talent identification process, and the corresponding need for identification methods/instruments that comprehensively target these characteristics, (b) deliberate attempts at the identification of predictors of future success among these diverse performance characteristics, (c) a stronger understanding of some confounding issues in the identification process (i.e., biological maturity and the relative age effect), and the investigation of possible solutions to such issues (e.g., bio-banding), (d) the development and validation of a number of non-sports specific and sports-specific identification instruments with sound psychometric properties, and (e) the establishment of specific features of optimal talent development interventions, that encompass both “on field” and “off field” interventions, for various sports. Of note, many of these advances relate to new areas of focus in the research in the area since 2015. Specifically, many of the studies that highlight the importance of multidimensional characteristics in identification have been recent studies (Aujla et al., 2015; Woods et al., 2016b; Turner et al., 2017; Scharfen and Memmert, 2019; Bennett et al., 2020; Towlson et al., 2022), while most studies that investigated performance characteristics which may be predictive of future success at the senior level have been conducted since 2015 (Deprez et al., 2015; Pion et al., 2015a; Till et al., 2017; Craig and Swinton, 2020; Cripps et al., 2020; Datson et al., 2020; Dugdale et al., 2021; Patel et al., 2021). Moreover, the confounding effects of biological maturity and the relative age effect have been a greater focus of the research over the past 5 years than in earlier periods (Andronikos et al., 2016; Furley and Memmert, 2016; Gorski et al., 2016; Myburgh et al., 2016; Till et al., 2017; Towlson et al., 2017; Peña-González et al., 2018, 2021; Lovell et al., 2019; Toum et al., 2021).

There appears to be a particular depth and breadth in the research on the anthropometric and physical performance characteristics, in comparison to the other performance characteristics, of gifted/talented young athletes across multiple sports. Relatedly, there appears to be considerable depth in the approaches and instruments that may be used to identify anthropometric and physical performance characteristics of these athletes. One possible reason for such a focus may be the fact that such characteristics are more readily, objectively, and reliably assessed with established, psychometrically rigorous instruments, than other characteristics that may involve some subjectivity in assessment (Prieto-Ayuso et al., 2022). A second contributing factor may be the possible bias toward, or over-reliance on, anthropometric and physical performance characteristics in the current thinking about identification and development of physical giftedness. As an example, Bennett et al. (2020) noted a greater emphasis on physical maturity than on technical and tactical characteristics in the National Football Curriculum developed by Football Federation Australia.

Unsurprisingly, there is also substantial breadth and depth in the research on the two phenomena that may have added significance when the focus of identification processes is on anthropometric and physical performance characteristics—biological maturation and the relative age effect. The phenomena have been studied across multiple sports, and knowledge has advanced to such an extent that there is now recognition of sports where such phenomena are not significant, and even sports where a “reverse” relative age effect may be seen (e.g., sports that emphasize technical aspects and are not as reliant on biological maturation, such as golf, shooting, figure skating, and gymnastics Coutts et al., 2014). While multiple proposals have been made to address issues relating to biological maturation and the relative age effect in the identification process, research on the operationalization and implementation of such proposals appears to be less advanced.

Possibly related to the focus on anthropometric and physical performance characteristics may be the possibly *short-term* outlook in current talent identification processes across many sports. Even when non-anthropometric/physical performance characteristics are also assessed, a precedence often appears to be given to current performance over potential for future performance during identification procedures. For example, Cripps et al. (2020) noted that “selection at each level is determined by athlete attributes most likely to enhance performance at the specific developmental stage and not necessarily considerate of qualities likely to enhance adult performance or professional career attainment” (p. 507), while Bidaurrazaga-Letona et al. (2019) noted that “identification was based more on the current performance of players than on their future potential” (p. 2557). While such thinking may be grounded in the view that performance in early development may be indicative of future performance (Baker et al., 2017; Schorer et al., 2017), this may also be an overly simplistic perspective that does not give adequate consideration to the non-linear nature of developmental trajectories (Vandorpe et al., 2012). It may also reflect the many complexities and difficulties that are associated with the identification of future potential, including the lack of firmly established procedures and instruments. Irrespective of the precise reasons, an encouraging recent development is the focus of some of the latest research in the area on the identification of significant predictors of future professional success across sports.

As a complement to the research on the identification of giftedness/talent, is a growing body of empirical research on talent development interventions, which collectively outline some key features of the “physical” and “non-physical” components of successful programs and provisions in various sports. While some of the research has investigated generic interventions that may have application for gifted/talented individuals across multiple physical domains, much of the research appears to be specific to individual physical domains. Furthermore, much of the scholarly attention appears to be focused on summer and team sports, over winter and individual sports. The fact that most of these studies have been conducted in connection to talent development practices outside of school settings, suggests that much remains unknown about effective practices to support gifted/talented athletes who do not have access to external talent development opportunities.

### Areas for future investigation

This review of the literature on physical giftedness/talent has identified a number of areas for focus in future research. First of all, there is a need for the continuation of the encouraging trends in the rising volume of quality research in the area. Within the period under investigation, the average number of publications that met the inclusion/exclusion criteria has risen from 0.4 publications per year in 2000–2004 to 2 publications per year in 2005–2009, 5.2 publications per year in 2010–2014, 8.6 publications per year in 2015–2019, to 10 publications per year in 2020 and 2021. A continuation of this growth, within different contexts, that build on and/or replicate prior studies, is likely to be conducive to a greater comprehensiveness of the state of knowledge in the area, greater generalizability of the findings, and greater confidence in the choice and implementation of identification/development practices.

Ideally, some of these future studies should be in specific targeted areas. One of these relates to the conceptions and theoretical bases for giftedness and talent in the physical domain. While some discourse exists on how to define and identify those who are gifted or talented in the physical domain (Abbott and Collins, 2002, 2004; Gagné, 2003; Bailey and Morley, 2006; Croston, 2013), there appears to be minimal empirical basis for such investigations. Furthermore, most empirical studies on the identification and development of physical giftedness/talent appear to neglect or ignore conceptual and/or theoretical frameworks on giftedness or talent. Instead, many authors omit any explicit statements of definitions or conceptions of giftedness/talent, or make the assumption that they are commonly understood. Such a lack of attention to the fundamental concepts that define the area is potentially problematic for theoretical development. As noted by Tranckle and Cushion (2006), “any future research efforts will be hampered until some consensus is reached over these issues” (p. 278). Some of the obvious benefits of having clearer conceptual and theoretical foundations for research in the area include an enhanced comparability of research, greater opportunities for the findings of one study to build on others, and a greater systematicity of investigations.

A second area for future research attention relates to the need for more multidimensional perspectives in the identification of giftedness/talent in the physical domain. Despite some encouraging recent trends in this direction, the bulk of the research in the area remains focused on the assessment of anthropometric and physical performance characteristics of young gifted/talented athletes. Among the various performance characteristics, there appears to be a particular need for attention to the assessment of sports-specific/technical, motor co-ordination, cognitive, perceptual, tactical, psychological, and sociological characteristics in the identification process to allow for a more comprehensive assessment of physical giftedness/talent (Hendry et al., 2018; Dugdale et al., 2021; Patel et al., 2021; Farley et al., 2022). The collective assessment of such characteristics (rather than the assessment of any of these characteristics in isolation), in addition to anthropometric and physical performance characteristics, may allow for a more informed and holistic approach to identification, that better reflects the multiple and complex contributors to success at the senior level (Reilly et al., 2000; Phillips et al., 2014; Woods et al., 2016b; Dugdale et al., 2021; Farley et al., 2022). A multidimensional approach to identification is also likely to be a fairer approach that may minimize the premature loss of talent (Patel et al., 2021). It is noteworthy that a multidimensional approach to identification has been strongly promoted in the field of gifted education due to the reduction in measurement errors, the inclusiveness of those from diverse backgrounds, and the provision of multiple opportunities for the demonstration of giftedness or talent (Hartas et al., 2008; Acar et al., 2016; Geiser et al., 2016; Cao et al., 2017).

As an important component of any comprehensive multidimensional approach to giftedness/talent identification, there is a need for an explicit acknowledgment of the subjective considerations by experts in the identification process. Although multiple scholars recognize that identification decisions are often made by experienced experts and coaches, on the basis of factors that may include their subjective appraisals of athletes along with objective assessments of performance characteristics (Ulbricht et al., 2016; Schorer et al., 2017; Woods et al., 2017; Hendry et al., 2018; Lovell et al., 2019), minimal research attention has so far been devoted to the factors and dynamics associated with such subjective appraisals. Greater clarity in how specifically such subjective considerations contribute to the talent identification process may allow for a more complete understanding of current identification processes, allow for evaluations of their suitability, and inform any measures to enhance such processes.

Any multidimensional approach to identification will necessitate a mechanism for optimally *combining* the identification data obtained from the various identification approaches/instruments, some of which may or may not contradict one other. While little attention has been devoted to the issue in the literature on the identification of giftedness/talent in the physical domain, some partial guidance exists from the field of gifted education. Specifically, Acar et al. (2016) suggest that data should be concurrently collected using both quantitative and qualitative assessments, while McBee et al. (2014) suggest that the data obtained from the different approaches/instruments should have reasonable levels of reliability, and fair levels of correlation with one another. Furthermore, to deal with inconsistent data from the various identification approaches/instruments, McBee et al. (2014) have proposed some combination rules—the conjunctive (“and”) rule that requires a minimum standard to be met on all identification approaches/methods, the disjunctive (“or”) rule that requires a minimum standard to be met on only one identification approach/method, and the compensatory (“mean”) rule that represents a compromise between the conjunctive and disjunctive rules. Different rules may have applicability depending on the level of selectivity that is required and the planned development interventions. Nevertheless, further research will be necessary to ascertain how applicable these guidelines may be to the identification of giftedness/talent in the physical domain.

Related to the optimal approaches for the combination of identification data from multiple sources, is a need for research that examines the interaction between the various performance characteristics of young gifted/talented athletes. Such research may be particularly useful in informing the applicability of various combination rules with respect to inconsistent identification data. At the present time, research on the topic is only at an emergent stage. Specifically, Scharfen and Memmert (2019) outlined a possible correlation between cognitive skills (i.e., attention window and working memory) and some soccer specific motor skills, while Turner et al. (2017) identified a relationship between anthropometric characteristics (i.e., arm and leg span) and the “reachability” of lunging and step-lunging attacks in fencing. Additional research will be useful with respect to the full range of performance characteristics across the various physical domains. Furthermore, greater attention will be useful on the possible *compensatory effects* of the various characteristics, which have been identified by Pion et al. (2015c) to be a factor that may influence talent development in gymnastics, and advocated by Abbott and Collins (2004) in their proposal to better identify and support future potential in sport.

As for the identification of giftedness/talent in the physical domain, a number of gaps appear to exist in the current knowledge on the development of giftedness/talent in the physical domain. That is, the somewhat non-systematic approach to research in the area may not be conducive to a holistic or a complete picture being gained on the optimal approaches to the development of giftedness/talent. Furthermore, some of the work in the area comprise conceptually-derived proposals for interventions that are non-empirical in nature (Abbott and Collins, 2004; Bailey and Morley, 2006; Baker et al., 2019). Some specific investigations that may be particularly useful to move the area forward include the empirical testing of the many conceptually derived proposals, the development of intervention options that may be applied in school settings where large numbers of gifted/talented athletes may have access, the development of a wider range of intervention options that are useful across sports, the matching of interventions to gifted/talented athletes of different characteristic profiles, the development of variations to current gifted/talent development interventions for athletes who display different levels of giftedness/talent, and the development of greater numbers of “off field” interventions to optimally support gifted/talented athletes. Ideally, systematic and integrated programs of research addressing these issues should be pursued.

Apart from the specific areas that are in need of future research attention, this systematic review of the literature has identified a number of possible directions in the *manner* in which future studies could be conducted. Over the investigated period, there has been no substantial variation in the breakdown of quantitative/qualitative studies, methods of data collection, or methods of data analysis. That is, most studies have been quantitative in nature, involve the collection of measurements of various performance characteristics, and involve analysis using general linear model analytical techniques (i.e., ANOVA, ANCOVA, MANOVA, MANCOVA, ordinary linear regression, *t*-tests). As such, there may be some benefit in introducing greater diversity in the methodology. Perhaps greater numbers of longitudinal studies may be useful, as the research on identification is often limited by the cross-sectional nature of the methodologies that have traditionally been adopted. While these methodologies have merit, they tend to assume that current adolescent performance may be used to predict outcomes in adulthood, and fail to consider the often dynamic and non-linear nature of development (Till et al., 2017; Hendry et al., 2018; Dugdale et al., 2021). Furthermore, there may be some benefit in the greater adoption of qualitative and mixed methods approaches to research, to allow for alternative perspectives to the general pattern of existing findings to emerge. Some obvious areas that may be suited to qualitative study include the subjective appraisals of gifted/talented athletes and conceptual/theoretical work on giftedness and talent in the physical domain.

Finally, it is noted that the vast majority of the studies that were selected for the systematic review of the literature were published in peer reviewed outlets in the field of sport sciences, probably reflecting the sport science focus of most existing studies relevant to giftedness and talent in the physical domain. Only a small number of studies have been published in the fields of psychology and education. Consequently, much of the research on physical giftedness and talent may be somewhat devoid of exposure to the many ideas, findings, and practices outside of sports science. Such “insularity” in the research literature may limit the development and advancement of the area. More extensive collaboration is therefore encouraged with scholars in diverse fields, including scholars in the field of gifted education who have a shared interest in the identification and development of exceptional ability and achievement.

## Data availability statement

The original contributions presented in the study are included in the article/Supplementary material, further inquiries can be directed to the corresponding author/s.

## Author contributions

The author confirms being the sole contributor of this work and has approved it for publication.

## Funding

This work was supported by the New South Wales Department of Education under Grant RG212886.

## Conflict of interest

The author declares that the research was conducted in the absence of any commercial or financial relationships that could be construed as a potential conflict of interest.

## Publisher's note

All claims expressed in this article are solely those of the authors and do not necessarily represent those of their affiliated organizations, or those of the publisher, the editors and the reviewers. Any product that may be evaluated in this article, or claim that may be made by its manufacturer, is not guaranteed or endorsed by the publisher.
