# Supplementary material for: Physical giftedness/talent: A systematic review of the literature on identification and development
Source: Front Psychol. 2022 Aug 26;13:961624. doi: 10.3389/fpsyg.2022.961624 (PMC9460770; doi:10.3389/fpsyg.2022.961624)
Supplement: Supplementary file 1 [file Data_Sheet_1.docx]

**Articles selected for the systematic literature review**

Andronikos, G., Elumaro, A. I., Westbury, T., & Martindale, R. J. (2016). Relative age effect: Implications for effective practice. *Journal of Sports Sciences*, *34*(12), 1124–1131. https://doi.org/10.1080/02640414.2015.1093647

Aouadi, R., Jlid, M. C., Khalifa, R., Hermassi, S., Chelly, M. S., Van Den Tillaar, R., & Gabbett, T. (2012). Association of anthropometric qualities with vertical jump performance in elite male volleyball players. *The Journal of Sports Medicine and Physical Fitness*, *52*(1), 11–17. https://doi.org/10.4100/jhse.2012.73.11

Arede, J., Esteves, P., Ferreira, A. P., Sampaio, J., & Leite, N. (2019). Jump higher, run faster: effects of diversified sport participation on talent identification and selection in youth basketball. *Journal of Sports Sciences*, *37*(19), 2220–2227. https://doi.org/10.1080/02640414.2019.1626114

Aujla, I. J., Nordin-Bates, S. M., & Redding, E. (2015). Multidisciplinary predictors of adherence to contemporary dance training: Findings from the UK Centres for Advanced Training. *Journal of Sports Sciences*, *33*(15), 1564–1573. https://doi.org/10.1080/02640414.2014.996183

Bennett, K. J., Novak, A. R., Pluss, M. A., Coutts, A. J., & Fransen, J. (2020). A multifactorial comparison of Australian youth soccer players’ performance characteristics. *International Journal of Sports Science & Coaching*, *15*(1), 17–25. https://doi.org/10.1177/1747954119893174

Bidaurrazaga-Letona, I., Lekue, J. A., Amado, M., & Gil, S. M. (2019). Progression in youth soccer: Selection and identification in youth soccer players aged 13–15 years. *The Journal of Strength & Conditioning Research*, *33*(9), 2548–2558. https://doi.org/10.1519/JSC.0000000000001924

Bonney, N., Berry, J., Ball, K., & Larkin, P. (2020). Validity and reliability of an Australian football small-sided game to assess kicking proficiency. *Journal of Sports Sciences*, *38*(1), 79–85. https://doi.org/10.1080/02640414.2019.1681864

Buchheit, M., & Mendez-Villanueva, A. (2013). Reliability and stability of anthropometric and performance measures in highly-trained young soccer players: Effect of age and maturation. *Journal of Sports Sciences*, *31*(12), 1332–1343. https://doi.org/10.1080/02640414.2013.781662

Burgess, D., Naughton, G., & Hopkins, W. (2012). Draft-camp predictors of subsequent career success in the Australian Football League. *Journal of Science and Medicine in Sport*, *15*(6), 561–567. https://doi.org/10.1016/j.jsams.2012.01.006

Burr, J. F., Jamnik, R. K., Baker, J., Macpherson, A., Gledhill, N., & McGuire, E. J. (2008). Relationship of physical fitness test results and hockey playing potential in elite-level ice hockey players. *The Journal of Strength & Conditioning Research*, *22*(5), 1535–1543. https://doi.org/10.1519/JSC.0b013e318181ac20

Castagna, C., Manzi, V., Impellizzeri, F., Weston, M., & Alvarez, J. C. B. (2010). Relationship between endurance field tests and match performance in young soccer players. *The Journal of Strength & Conditioning Research*, *24*(12), 3227–3233. 10.1519/JSC.0b013e3181e72709

Collins, D., Martindale, R., Button, A., & Sowerby, K. (2010). Building a physically active and talent rich culture: An educationally sound approach. *European Physical Education Review*, *16*(1), 7–28. https://doi.org/10.1177/1356336X10369196

Coutts, A. J., Kempton, T., & Vaeyens, R. (2014). Relative age effects in Australian Football League national draftees. *Journal of Sports Sciences*, *32*(7), 623–628. https://doi.org/10.1080/02640414.2013.847277

Craig, T. P., & Swinton, P. (2020). Anthropometric and physical performance profiling does not predict professional contracts awarded in an elite Scottish soccer academy over a 10-year period. *European Journal of Sport Science, 21*(8), 1101–1110. https://doi.org/10.1080/17461391.2020.1808079

Cripps, A. J., Banyard, H. G., Woods, C. T., Joyce, C., & Hopper, L. S. (2020). Does the longitudinal development of physical and anthropometric characteristics associate with professional career attainment in adolescent Australian footballers? *International Journal of Sports Science & Coaching*, *15*(4), 506–511. https://doi.org/10.1177/1747954120927117

Croston, A. (2013). “A clear and obvious ability to perform physical activity”: Revisiting physical education teachers' perceptions of talent in PE and sport. *Physical Education and Sport Pedagogy, 18*(1), 60–74. <https://doi.org/10.1080/17408989.2011.631001>

Datson, N., Weston, M., Drust, B., Gregson, W., & Lolli, L. (2020). High-intensity endurance capacity assessment as a tool for talent identification in elite youth female soccer. *Journal of Sports Sciences, 38*(11–12), 1313–1319. <https://doi.org/10.1080/02640414.2019.1656323>

Deprez, D. N., Fransen, J., Lenoir, M., Philippaerts, R. M., & Vaeyens, R. (2015). A retrospective study on anthropometrical, physical fitness, and motor coordination characteristics that influence dropout, contract status, and first-team playing time in high-level soccer players aged eight to eighteen years. *The Journal of Strength & Conditioning Research*, *29*(6), 1692–1704. https://doi.org/10.1519/JSC.0000000000000806

De Siati, F., Laffaye, G., Gatta, G., Dello Iacono, A., Ardigò, L. P., & Padulo, J. (2016). Neuromuscular and technical abilities related to age in water-polo players. *Journal of Sports Sciences*, *34*(15), 1466–1472. https://doi.org/10.1080/02640414.2015.1119298

Dobbin, N., Highton, J., Moss, S. L., & Twist, C. (2019). The discriminant validity of a standardized testing battery and its ability to differentiate anthropometric and physical characteristics between youth, academy, and senior professional rugby league players. *International Journal of Sports Physiology and Performance*, *14*(8), 1110–1116. https://doi.org/10.1123/ijspp.2018-0519

Dugdale, J. H., Sanders, D., Myers, T., Williams, A. M., & Hunter, A. M. (2020). A case study comparison of objective and subjective evaluation methods of physical qualities in youth soccer players. *Journal of Sports Sciences*, *38*(11-12), 1304–1312. https://doi.org/10.1080/02640414.2020.1766177

Dugdale, J. H., Sanders, D., Myers, T., Williams, A. M., & Hunter, A. M. (2021). Progression from youth to professional soccer: A longitudinal study of successful and unsuccessful academy graduates. *Scandinavian Journal of Medicine & Science in Sports*, *31*(S1), 73–84. https://doi.org/10.1111/sms.13701

Falk, B., Lidor, R., Lander, Y., & Lang, B. (2004). Talent identification and early development of elite water-polo players: A 2-year follow-up study. *Journal of Sports Sciences*, *22*(4), 347–355. https://doi.org/10.1080/02640410310001641566

Farley, J. B., Keogh, J. W. L., Woods, C. T., & Milne, N. (2021). Physical fitness profiles of female Australian football players across five competition levels. *Science and Medicine in Football.* Advance online publication. https://doi.org/10.1080/24733938.2021.1877335

Fontana, F. Y., Colosio, A., De Roia, G. F., Da Lozzo, G., & Pogliaghi, S. (2015). Anthropometrics of Italian senior male rugby union players: From elite to second division. *International Journal of Sports Physiology and Performance*, *10*(6), 674–680. https://doi.org/10.1123/ijspp.2015-0014

Furley, P., & Memmert, D. (2016). Coaches’ implicit associations between size and giftedness: implications for the relative age effect. *Journal of Sports Sciences*, *34*(5), 459–466. https://doi.org/10.1080/02640414.2015.1061198

Gabbett, T., Georgieff, B., Anderson, S., Cotton, B., Savovic, D., & Nicholson, L. (2006). Changes in skill and physical fitness following training in talent-identified volleyball players. *The Journal of Strength & Conditioning Research*, *20*(1), 29–35. https://doi.org/10.1519/00124278-200602000-00005

Galy, O., Zongo, P., Chamari, K., Chaouachi, A., Michalak, E., Dellal, A., ... & Hue, O. (2015). Anthropometric and physiological characteristics of Melanesian futsal players: a first approach to talent identification in Oceania. *Biology of Sport*, *32*(2), 135–141. https://doi.org/10.5604/20831862.1140428

Gaudion, S. L., Doma, K., Sinclair, W., Banyard, H. G., & Woods, C. T. (2017). Identifying the physical fitness, anthropometric and athletic movement qualities discriminant of developmental level in elite junior Australian football: Implications for the development of talent. *The Journal of Strength & Conditioning Research*, *31*(7), 1830–1839. https://doi.org/10.1519/JSC.0000000000001682

Gil, S. M., Zabala-Lili, J., Bidaurrazaga-Letona, I., Aduna, B., Lekue, J. A., Santos-Concejero, J., & Granados, C. (2014). Talent identification and selection process of outfield players and goalkeepers in a professional soccer club. *Journal of Sports Sciences*, *32*(20), 1931–1939. https://doi.org/10.1080/02640414.2014.964290

Gonçalves, C. E., Carvalho, H. M., & Diogo, F. L. (2014). A multilevel approach to the path to expertise in three different competitive settings. *Journal of Sports Science & Medicine*, *13*(1), 166–171.

Gorski, T., Rosser, T., Hoppeler, H., & Vogt, M. (2016). Relative age effect in young Swiss alpine skiers from 2004 to 2011. *International Journal of Sports Physiology and Performance*, *11*(4), 455–463. https://doi.org/10.1123/ijspp.2014-0418

Hay, P. J., & Macdonald, D. (2010). Evidence for the social construction of ability in physical education. *Sport, Education and Society*, *15*(1), 1–18. https://doi.org/10.1080/13573320903217075

Helsen, W. F., Van Winckel, J., & Williams, A. M. (2005). The relative age effect in youth soccer across Europe. *Journal of Sports Sciences*, *23*(6), 629–636. https://doi.org/10.1080/02640410400021310

Hendry, D. T., Williams, A. M., & Hodges, N. J. (2018). Coach ratings of skills and their relations to practice, play and successful transitions from youth-elite to adult-professional status in soccer. *Journal of Sports Sciences*, *36*(17), 2009–2017. https://doi.org/10.1080/02640414.2018.1432236

Hill, A., MacNamara, Á., Collins, D., & Rodgers, S. (2016). Examining the role of mental health and clinical issues within talent development. *Frontiers in Psychology*, *6*, 2042. https://doi.org/10.3389/fpsyg.2015.02042

Hogarth, L., Nicholson, V., Payton, C., & Burkett, B. (2021). Modelling the age‐related trajectory of performance in Para swimmers with physical, vision and intellectual impairment. *Scandinavian Journal of Medicine and Science in Sports*, *31*(4), 925–935. https://doi.org/10.1111/sms.13910

Holt, J. E., Kinchin, G., & Clarke, G. (2012). Effects of peer-assessed feedback, goal setting and a group contingency on performance and learning by 10–12-year-old academy soccer players. *Physical Education & Sport Pedagogy*, *17*(3), 231–250. https://doi.org/10.1080/17408989.2012.690568

Kegelaers, J., Wylleman, P., & Oudejans, R. R. (2020). A coach perspective on the use of planned disruptions in high-performance sports. *Sport, Exercise, and Performance Psychology*, *9*(1), 29–44. <https://doi.org/10.1037/spy0000167>

Keogh, J. W., Weber, C. L., & Dalton, C. T. (2003). Evaluation of anthropometric, physiological, and skill-related tests for talent identification in female field hockey. *Canadian Journal of Applied Physiology*, *28*(3), 397–409. https://doi.org/10.1139/h03-029

Kilger, M., & Jonsson, R. (2017). Talent production in interaction: Performance appraisal interviews in talent selection camps. *Communication & Sport*, *5*(1), 110–129. https://doi.org/10.1177/2167479515591789

Kramer, T., Huijgen, B. C., Elferink-Gemser, M. T., & Visscher, C. (2016). A longitudinal study of physical fitness in elite junior tennis players. *Pediatric Exercise Science*, *28*(4), 553–564. https://doi.org/10.1123/pes.2016-0022

Krüger, K., Pilat, C., Ückert, K., Frech, T., & Mooren, F. C. (2014). Physical performance profile of handball players is related to playing position and playing class. *The Journal of Strength & Conditioning Research*, *28*(1), 117–125. https://doi.org/10.1519/JSC.0b013e318291b713

Lamb, P., & Aldous, D. (2014). The role of e-mentoring in distinguishing pedagogic experiences of gifted and talented pupils in physical education. *Physical Education and Sport Pedagogy*, *19*(3), 301–319. https://doi.org/10.1080/17408989.2012.761682

Lidor, R., Falk, B., Arnon, M., & Cohen, Y. (2005). Measurement of talent in team handball: the questionable use of motor and physical tests. *The Journal of Strength and Conditioning Research*, *19*(2), 318–325. https://doi.org/10.1519/00124278-200505000-00014

Lidor, R., Hershko, Y., Bilkevitz, A., Arnon, M., & Falk, B. (2007). Measurement of talent in volleyball: 15-month follow-up of elite adolescent players. *Journal of Sports Medicine and Physical Fitness*, *47*(2), 159–168.

Lidor, R., Melnik, Y., Bilkevitz, A., Arnon, M., & Falk, B. (2005). Measurement of talent in judo using a unique, judo-specific ability test. *Journal of Sports Medicine and Physical Fitness*, *45*(1), 32–37.

Lovell, R., Fransen, J., Ryan, R., Massard, T., Cross, R., Eggers, T., & Duffield, R. (2019). Biological maturation and match running performance: A national football (soccer) federation perspective. *Journal of Science and Medicine in Sport*, *22*(10), 1139–1145. https://doi.org/10.1016/j.jsams.2019.04.007

MacNamara, Á., & Collins, D. (2011). Development and initial validation of the psychological characteristics of developing excellence questionnaire. *Journal of Sports Sciences*, *29*(12), 1273–1286. https://doi.org/10.1080/02640414.2011.589468

Matthys, S. P., Vaeyens, R., Fransen, J., Deprez, D., Pion, J., Vandendriessche, J., ... & Philippaerts, R. (2013). A longitudinal study of multidimensional performance characteristics related to physical capacities in youth handball. *Journal of Sports Sciences, 31*(3), 325–334. https://doi.org/10.1080/02640414.2012.733819

Matthys, S.P., Vaeyens, R., Vandendriessche, J., Vandorpe, B., Pion, J., Coutts, A.J., Lenoir, M. & Philippaerts, R.M. (2011). A multidisciplinary identification model for youth handball. *European Journal of Sport Science, 11*(5), 355–363. https://doi.org/10.1080/17461391.2010.523850

McKenzie, C. R., Whatman, C., & Brughelli, M. (2020). Performance profiling of female youth netball players. *The Journal of Strength & Conditioning Research*, *34*(11), 3275–3283. https://doi.org/10.1519/JSC.0000000000002958

Milić, M., Grgantov, Z., Chamari, K., Ardigò, L. P., Bianco, A., & Padulo, J. (2017). Anthropometric and physical characteristics allow differentiation of young female volleyball players according to playing position and level of expertise. *Biology of Sport*, *34*(1), 19–26. https://doi.org/10.5114/biolsport.2017.63382

Mkaouer, B., Hammoudi-Nassib, S., Amara, S., & Chaabène, H. (2018). Evaluating the physical and basic gymnastics skills assessment for talent identification in men’s artistic gymnastics proposed by the International Gymnastics Federation. *Biology of Sport*, *35*(4), 383–392. https://doi.org/10.5114/biolsport.2018.78059

Mohamed, H., Vaeyens, R., Matthys, S., Multael, M., Lefevre, J., Lenoir, M., & Philippaerts, R. (2009). Anthropometric and performance measures for the development of a talent detection and identification model in youth handball. *Journal of Sports Sciences, 27*(3), 257–266. https://doi.org/10.1080/02640410802482417

Moss, S. L., McWhannell, N., Michalsik, L. B., & Twist, C. (2015). Anthropometric and physical performance characteristics of top-elite, elite and non-elite youth female team handball players. *Journal of Sports Sciences*, *33*(17), 1780–1789. https://doi.org/10.1080/02640414.2015.1012099

Mostaert, M., Deconinck, F., Pion, J., & Lenoir, M. (2016). Anthropometry, physical fitness and coordination of young figure skaters of different levels. *International Journal of Sports Medicine*, *37*(7), 531–538. https://doi.org/10.1055/s-0042-100280

Myburgh, G. K., Cumming, S. P., Silva, M. C. E., Cooke, K., & Malina, R. M. (2016). Maturity-associated variation in functional characteristics of elite youth tennis players. *Pediatric Exercise Science, 28*(4), 542–552. https://doi.org/10.1123/pes.2016-0035

Nassib, S. H., Mkaouer, B., Riahi, S. H., Wali, S. M., & Nassib, S. (2020). Prediction of gymnastics physical profile through an international program evaluation in women artistic gymnastics. *The Journal of Strength & Conditioning Research*, *34*(2), 577–586. https://doi.org/10.1519/JSC.0000000000001902

Nikolaidis, P. T., Ingebrigtsen, J., Póvoas, S. C., Moss, S., Torres-Luque, G., & Pantelis, N. (2015). Physical and physiological characteristics in male team handball players by playing position: Does age matter. *Journal of Sports Medicine and Physical Fitness*, *55*(4), 297–304.

Norjali, R., Mostaert, M., Pion, J., & Lenoir, M. (2018). Anthropometry, physical performance, and motor coordination of medallist and non-medallist young fencers. *Archives of Budo*, *14*, 33–40.

Pastor-Vicedo, J. C., Prieto-Ayuso, A., Contreras-Jordán, O. R., Clemente, F. M., Nikolaidis, P. T., Rosemann, T. J., & Knechtle, B. (2020). Teaching and learning process of decision-making units in talented young players from U-10 to U-14. *Frontiers in Psychology*, *11*, 600. https://doi.org/10.3389/fpsyg.2020.00600

Patel, R., Nevill, A., Smith, T., Cloak, R., & Wyon, M. (2021). The influence of birth quartile, maturation, anthropometry and physical performances on player retention: Observations from an elite football academy. *International Journal of Sports Science & Coaching, 15*(2), 121–134. https://doi.org/10.1177/1747954120906507

Pearce, L. A., Sinclair, W. H., Leicht, A. S., & Woods, C. T. (2018). Physical, anthropometric, and athletic movement qualities discriminate development level in a rugby league talent pathway. *The Journal of Strength & Conditioning Research*, *32*(11), 3169–3176. https://doi.org/10.1519/JSC.0000000000002350

Peña-González, I., Fernández-Fernández, J., Moya-Ramón, M., & Cervelló, E. (2018). Relative age effect, biological maturation, and coaches’ efficacy expectations in young male soccer players. *Research Quarterly for Exercise and Sport*, *89*(3), 373–379. https://doi.org/10.1080/02701367.2018.1486003

Peña-González, I., García-Calvo, T., Cervelló, E. M., & Moya-Ramón, M. (2021). The coaches’ efficacy expectations of youth soccer players with different maturity status and physical performance. *Journal of Human Kinetics, 79*(1), 289–299. https://doi.org/10.2478/hukin-2021-0083

Phillips, E., Davids, K., Renshaw, I., & Portus, M. (2014). Acquisition of expertise in cricket fast bowling: Perceptions of expert players and coaches. *Journal of Science and Medicine in Sport*, *17*(1), 85–90. https://doi.org/10.1016/j.jsams.2013.03.005

Pion, J. A., Fransen, J., Deprez, D. N., Segers, V. I., Vaeyens, R., Philippaerts, R. M., & Lenoir, M. (2015). Stature and jumping height are required in female volleyball, but motor coordination is a key factor for future elite success. *The Journal of Strength & Conditioning Research, 29*(6), 1480–1485. <https://doi.org/10.1519/JSC.0000000000000778>

Pion, J., Lenoir, M., Vandorpe, B., & Segers, V. (2015). Talent in female gymnastics: a survival analysis based upon performance characteristics. *International Journal of Sports Medicine*, *94*(11), 935–940. https://doi.org/10.1055/s-0035-1548887

Pion, J. A., Segers, V., Fransen, J., Debuyck, G., Deprez, D., Haerens, L., Vaeyens, R., Philippaerts, R., & Lenoir, M. (2015). Generic anthropometric and performance characteristics among elite adolescent boys in nine different sports. *European Journal of Sport Science*, *15*(5), 357–366. <https://doi.org/10.1080/17461391.2014.944875>

Prieto-Ayuso, A., León, M. P., Contreras-Jordán, O. R., & Morley, D. (2021). Spanish physical education teachers’ perceptions of talent development. *European Physical Education Review.* Advance online publication. https://doi.org/10.1177/1356336X211010838

Ransdell, L. B., & Murray, T. (2011). A physical profile of elite female ice hockey players from the USA. *The Journal of Strength & Conditioning Research*, *25*(9), 2358–2363. https://doi.org/10.1519/JSC.0b013e31822a5440

Ransdell, L. B., Murray, T. M., & Gao, Y. (2013). Off-ice fitness of elite female ice hockey players by team success, age, and player position. *The Journal of Strength & Conditioning Research*, *27*(4), 875–884. https://doi.org/10.1519/JSC.0b013e3182651fd2

Robertson, K., Pion, J., Mostaert, M., Norjali Wazir, M. R. W., Kramer, T., Faber, I. R., Vansteenkiste, P., & Lenoir, M. (2018). A coaches’ perspective on the contribution of anthropometry, physical performance, and motor coordination in racquet sports. *Journal of Sports Sciences*, *36*(23), 2706–2715. https://doi.org/10.1080/02640414.2018.1441941

Sandercock, G. R., Taylor, M. J., Voss, C., Ogunleye, A. A., Cohen, D. D., & Parry, D. A. (2013). Quantification of the relative age effect in three indices of physical performance. *The Journal of Strength & Conditioning Research*, *27*(12), 3293–3299. https://doi.org/10.1519/JSC.0b013e318291b28d

Scharfen, H.-E., & Memmert, D. (2019). The relationship between cognitive functions and sport specific motor skills in elite youth soccer players. *Frontiers in Psychology, 10*, 817. https://doi. org/10.3389/fpsyg.2019.00817

Schorer, J., Rienhoff, R., Fischer, L., & Baker, J. (2017). Long-term prognostic validity of talent selections: Comparing national and regional coaches, laypersons and novices. *Frontiers in Psychology*, *8*, 1146. https://doi.org/10.3389/fpsyg.2017.01146

Spathis, J. G., Connick, M. J., Beckman, E. M., Newcombe, P. A., & Tweedy, S. M. (2015). Reliability and validity of a talent identification test battery for seated and standing Paralympic throws. *Journal of Sports Sciences*, *33*(8), 863–871. https://doi.org/10.1080/02640414.2014.969294

Sullivan, C., Kempton, T., Ward, P., & Coutts, A. J. (2020). The efficacy of talent selection criteria in the Australian Football League. *Journal of Sports Sciences*, *38*(7), 773–779. https://doi.org/10.1080/02640414.2020.1734309

Suraci, B. R., Quigley, C., Thelwell, R. C., & Milligan, G. S. (2021). A comparison of training modality and total genotype scores to enhance sport-specific biomotor abilities in under 19 male soccer players. *The Journal of Strength & Conditioning Research*, *35*(1), 154–161. https://doi.org/10.1519/JSC.0000000000003299

Taylor, J., & Collins, D. (2019). Shoulda, coulda, didnae—Why don’t high-potential players make it? *The Sport Psychologist*, *33*(2), 85–96. https://doi.org/10.1123/tsp.2017-0153

te Wierike, S. C., de Jong, M. C., Tromp, E. J., Vuijk, P. J., Lemmink, K. A., Malina, R. M., ... & Visscher, C. (2014). Development of repeated sprint ability in talented youth basketball players. *The Journal of Strength & Conditioning Research*, *28*(4), 928–934. https://doi.org/10.1097/JSC.0000000000000223

Till, K., Cobley, S., O’Hara, J., Brightmore, A., Cooke, C., & Chapman, C. (2011). Using anthropometric and performance characteristics to predict selection in junior UK Rugby League players. *Journal of Science and Medicine in Sport*, *14*(3), 264–269. https://doi.org/10.1016/j.jsams.2011.01.006

Till, K., Morley, D., O’Hara, J., Jones, B. L., Chapman, C., Beggs, C. B., Cooke, C., & Cobley, S. (2017). A retrospective longitudinal analysis of anthropometric and physical qualities that associate with adult career attainment in junior rugby league players. *Journal of Science and Medicine in Sport, 20*(11), 1029–1033. https://doi.org/10.1016/j.jsams.2017.03.018

Toum, M., Tribolet, R., Watsford, M. L., & Fransen, J. (2020). The confounding effect of biological maturity on talent identification and selection within youth Australian football. *Science and Medicine in Football*, 1–9. <https://doi.org/10.1080/24733938.2020.1822540>

Towlson, C., Cobley, S., Midgley, A. W., Garrett, A., Parkin, G., & Lovell, R. (2017). Relative age, maturation and physical biases on position allocation in elite-youth soccer. *International Journal of Sports Medicine*, *38*(3), 201–209. https://doi.org/10.1055/s-0042-119029

Towlson, C., MacMaster, C., Gonçalves, B., Sampaio, J., Toner, J., MacFarlane, N., Barrett, S., Hamilton, A., Jack, R., Hunter, F., Myers, T., Abt, G. (2021). The effect of bio-banding on physical and psychological indicators of talent identification in academy soccer players. *Journal of Science and Medicine in Football*. Advance online publication. https://doi.org/10.1080/24733938.2020.1862419

Turner, A. N., Marshall, G., Noto, A., Chavda, S., Atlay, N., & Kirby, D. (2017). Staying out of range: Increasing attacking distance in fencing. *International Journal of Sports Physiology and Performance*, *12*(10), 1319–1323. https://doi.org/10.1123/ijspp.2016-0680

Ulbricht, A., Fernandez-Fernandez, J., Mendez-Villanueva, A., & Ferrauti, A. (2016). Impact of fitness characteristics on tennis performance in elite junior tennis players. *The Journal of Strength & Conditioning Research*, *30*(4), 989–998. https://doi.org/10.1519/JSC.0000000000001267

Vaeyens, R., Malina, R. M., Janssens, M., Van Renterghem, B., Bourgois, J., Vrijens, J., & Philippaerts, R. M. (2006). A multidisciplinary selection model for youth soccer: the Ghent Youth Soccer Project. *British Journal of Sports Medicine*, *40*(11), 928–934.

https://doi.org/10.1136/bjsm.2006.029652

Vandorpe, B., Vandendriessche, J. B., Vaeyens, R., Pion, J., Lefevre, J., Philippaerts, R. M., & Lenoir, M. (2012). The value of a non-sport-specific motor test battery in predicting performance in young female gymnasts. *Journal of Sports Sciences*, *30*(5), 497–505. https://doi.org/10.1080/02640414.2012.654399

Veale, J. P., Pearce, A. J., Koehn, S., & Carlson, J. S. (2008). Performance and anthropometric characteristics of prospective elite junior Australian footballers: a case study in one junior team. *Journal of Science and Medicine in Sport*, *11*(2), 227–230. https://doi.org/10.1016/j.jsams.2006.12.119

Vescovi, J. D. (2014). Motion characteristics of youth women soccer matches: Female Athletes in Motion (FAiM) Study. *International Journal of Sports Medicine*, *35*(2), 110–117. https://doi.org/10.1055/s-0033-1345134

Vescovi, J. D., Rupf, R., Brown, T. D., & Marques, M. C. (2011). Physical performance characteristics of high‐level female soccer players 12–21 years of age. *Scandinavian Journal of Medicine & Science in Sports*, *21*(5), 670–678. https://doi.org/10.1111/j.1600-0838.2009.01081.x

Votteler, A., & Höner, O. (2014). The relative age effect in the German Football TID Programme: Biases in motor performance diagnostics and effects on single motor abilities and skills in groups of selected players. *European Journal of Sport Science*, *14*(5), 433–442. https://doi.org/10.1080/17461391.2013.837510

Wells, G. D., Elmi, M., & Thomas, S. (2009). Physiological correlates of golf performance. *The Journal of Strength & Conditioning Research*, *23*(3), 741–750. https://doi.org/10.1519/JSC.0b013e3181a07970

Wood, D. J., Coughlan, G. F., & Delahunt, E. (2018). Fitness profiles of elite adolescent Irish rugby union players. *The Journal of Strength & Conditioning Research*, *32*(1), 105–112. https://doi.org/10.1519/JSC.0000000000001694

Woods, C. T., Bruce, L., Veale, J. P., & Robertson, S. (2016). The relationship between game-based performance indicators and developmental level in junior Australian football: Implications for coaching. *Journal of Sports Sciences*, *34*(23), 2165–2169. https://doi.org/10.1080/02640414.2016.1210816

Woods, C. T., Cripps, A., Hopper, L., & Joyce, C. (2017). A comparison of the physical and anthropometric qualities explanatory of talent in the elite junior Australian football development pathway. *Journal of Science and Medicine in Sport*, *20*(7), 684–688. https://doi.org/10.1016/j.jsams.2016.11.002

Woods, C. T., Raynor, A. J., Bruce, L., McDonald, Z., & Robertson, S. (2016). The application of a multi-dimensional assessment approach to talent identification in Australian football. *Journal of Sports Sciences, 34*(14), 1340–1345. <https://doi.org/10.1080/02640414.2016.1142668>

Znazen, H., Mejri, A., Touhami, I., Chtara, M., Siala, H., Ahmetov, I. I., Messaoud, T., Chamari, K., & Soussi, N. (2016). Genetic advantageous predisposition of angiotensin converting enzyme id polymorphism in Tunisian athletes. *The Journal of Sports Medicine and Physical Fitness, 56*(6), 724–730.
